# Risk factors of postoperative pancreatic fistula after distal pancreatectomy using a triple-row stapler

**DOI:** 10.1007/s00595-017-1554-2

**Published:** 2017-06-09

**Authors:** Hiromichi Kawaida, Hiroshi Kono, Mitsuaki Watanabe, Naohiro Hosomura, Hidetake Amemiya, Hideki Fujii

**Affiliations:** First Department of Surgery, Faculty of Medicine, University of Yamanashi, 1110 Shimokato, Chuo-shi, Yamanashi, 409-3898 Japan

**Keywords:** Triple-row stapler, Distal pancreatectomy, Pancreatic fistula

## Abstract

**Purpose:**

Postoperative pancreatic fistula (POPF) is one of the major complications in patients who undergo distal pancreatectomy (DP). Recently, dividing the pancreas by stapler is a commonly performed technique, however, POPF still occurs. Therefore, the purpose of this study was to investigate the risk factors for POPF after DP using a triple-row stapler.

**Methods:**

A total of 75 patients underwent DP using a triple-row stapler (Endo GIA™ Reloads with Tri-Staple™ Technology 60 mm; COVIDIEN, North Haven, CT, USA) at Yamanashi University from December 2012 to December 2016. The clinical risk factors for POPF after DP using a triple-row stapler were identified based on univariate and multivariate analyses.

**Results:**

Clinical POPF (ISGPF Grade B and C) was seen in 7 of 75 patients (9.3%). The body mass index (BMI) was significantly higher in the patients with POPF (26.8 ± 0.5 kg/m^2^) compared with the patients without POPF (21.4 ± 0.4 kg/m^2^; a cut-off value; 25.7 kg/m^2^). In addition, the patients with POPF were significantly younger than the patients without POPF (56.4 ± 5.6 vs 67.0 ± 1.5; a cut-off value was 57.0 years old).

**Conclusions:**

BMI and age were found to be significant risk factors for POPF after DP using a triple-row stapler.

## Introduction

Although perioperative management and operative techniques in pancreatic surgery have improved, post-operative pancreatic fistula (POPF) remains the most common complication after distal pancreatectomy (DP), occurring in from 5 to 32% of all cases [1–6] based on the International Study Group of Pancreatic Fistula (ISGPF) criteria [7]. POPF can be triggered by serious incidents such as intra-abdominal abscess and intra-abdominal arterial bleeding and it can be fatal. To prevent POPF, several techniques including hand-sewn closure [8–10], stapled closure [2, 3, 8, 11, 12], main pancreatic duct ligation [8, 10], bipolar scissors [13], the application of fibrin glue [14, 15], pancreaticoenteric anastomosis [9, 16, 17], mesh reinforcement of pancreatic transection [18–20], TachoSil^®^ patches [21], Teres ligament patch [22] have been reported. Although the superiority of stapler closure is not clear compared to the other techniques [3, 8, 12], the simple and easy stapler closure method is recently becoming one of the commonly performed techniques. In addition, this technique can close both the main pancreatic duct and the branch pancreatic duct at the same time.

Various reports have discussed the risk factors for POPF after DP, however, the specific risk factors for POPF after DP using a triple-row stapler remain to be clearly elucidated. Therefore, the purpose of this study was to investigate the risk factors for POPF after DP using a triple-row stapler.

## Patients and methods

### Patients

DP was performed on a total of 75 patients at Yamanashi University between December 2012 and December 2016. The pancreas was transected with a triple row stapler (Endo GIA™ Reloads with Tri-Staple™ Technology 60 mm; COVIDIEN, North Haven, CT, USA). They were classified into a POPF Grade B/C group and none or a POPF Grade A group, because POPF Grade A is not important regarding its clinical significance, whereas Grade B and C are important.

Data were collected prospectively and the clinicopathological features were reviewed from the electronic medical records. Among the patients included in this study, there were no cases with a past history of pancreatitis that affected the remnant pancreas. In addition, there were no patients who were re-hospitalized due to late-onset POPF and there were no postoperative deaths. To supplement the perioperative data, a review of the surgical and anesthetic charts of each patient was performed. The patient characteristics are shown in Table 1.

**Table 1:** Patient characteristics

	POPF Grade B/C (*n* = 7 9.3%)	None or POPF Grade A (*n* = 68 90.7%)	Univariate analysis *p*	Multivariate analysis	Cut-off index
Preoperative status					
Age	56.4 ± 5.6	67.0 ± 1.5	0.041	0.083	57
Sex (male/female)	5/2	34/34	0.286		
BMI (kg/m^2^)	26.8 ± 0.5	21.4 ± 0.4	0.0001	0.028	25.7
HbA1c (%)	5.8 ± 0.2	6.2 ± 0.1	0.282		
Histopathological diagnosis					
Pancreatic adenocarcinoma	2 (28.6%)	28 (41.2%)			
Intraductal papillary mucinous neoplasm	0	20 (29.4%)			
Pancreatic neuroendocrine tumor	3 (42.9%)	11 (16.2%)			
Mucinous cyst neoplasm	1 (14.3%)	2 (2.9%)			
Serous cyst adenoma	0	1 (1.5%)			
Solid-pseudopapillary neoplasm	0	1 (1.5%)			
Other diseases	1 (14.3%)	5 (7.4%)			
Benign disease/malignant disease	4/3	36/32	0.835		
Neoadjuvant none/NAC/NACRT	7/0/0	65/2/1			
Duration of in-hospital day	56.4 ± 9.7	14.9 ± 0.9	<0.0001		

*BMI* body mass index, *HbA1c* hemoglobin A1c, *NAC* neoadjuvant chemotherapy, *NACRT* neoadjuvant chemoradiotherapy, *POPF* defined based on ISGPF

### Surgical procedures

All surgeries were performed by a pancreatic surgeon with more than 15 years of experience. After dissection of the peripancreatic space, the pancreas was divided using Endo GIA™ Reloads with Tri-Staple™. The cartridge height of the stapler was selected according to the thickness of the pancreatic cutting line. The pancreatic thickness of the pancreatic cutting line was measured by intraoperative ultrasonography.

A black cartridge was used for a thickness of more than 11 mm, and a purple cartridge was used for a thickness of less than 10 mm. The closure jaw was clamped slowly and carefully over a period of 5 min, and the pancreas was cut little by little over 15 min for the purpose of carefully performing the parenchymal flattening technique and then applying the staples. The stapler was not released immediately after firing. A closed drain was placed near the stump of the remnant pancreas. Intraoperative data are shown in Table 2.

**Table 2:** Intraoperative findings

	POPF Grade B/C (*n* = 7)	None or POPF Grade A (*n* = 68)	Univariate analysis *p*
Intraoperative findings performed operation DP			
+Splenectomy (yes/no)	3/4	48/20	0.138
+Lymph node dissection (D0, 1/D2)	5/2	37/31	0.198
+Gastrectomy	0	4	
+Colectomy	0	2	
Laparoscopy (yes/no)	1/6	21/47	0.365
Operative time (min)	427.9 ± 44.8	369.7 ± 15.7	0.257
Blood loss (ml)	1084.0 ± 242.2	696.6 ± 79.0	0.137
RBC transfusion (yes/no)	0/7	7/61	
Thickness of the stump	14.9 ± 2.1	12.6 ± 0.4	0.139
Width of the stump	34.9 ± 2.7	30.3 ± 0.9	0.133

*DP* distal pancreatectomy, *RBC* red blood cells

### Perioperative management

The amylase level of the serum and drainage fluid was measured on postoperative days (POD) 1 and 3. Oral diet consumptions were started on POD4 in general. To prevent a bacterial infection, second generation cefem antibiotics were used either intraoperatively or for 3 days postoperatively. Prophylactic somatostatin analogues were not administered to prevent POPF. A drainage tube was removed at POD3 or 4 regardless of the amount of drainage fluid, when the drainage fluid was clear, thus indicating that no bacterial infection existed.

POPF was diagnosed according to the International Study Group of Pancreatic Fistula (ISGPF) definition [7].

### Statistical analysis

Data were expressed as the mean ± standard deviation. Patient characteristics and intraoperative and postoperative factors between the two groups were compared by Chi-square statistics, Fisher’s exact test, and the Mann–Whitney *U* test. Univariate and multivariate logical regression analyses were conducted to identify the independent risk factors for POPF. The optimal cutoff level of the age, body mass index (BMI), thickness of the stump, and the amylase level of drainage fluid on POD 3 to differentiate between POPF Grade B/C and none or POPF Grade A were determined by constructing a receiver operating characteristic curve. The statistical analyses were performed using the SPSS version 23.0 software program (SPSS Inc, Chicago, IL, USA). *p* values pf less than 0.05 were considered to be statistically significant.

## Results

### Patient characteristics

**Fig. 1:**
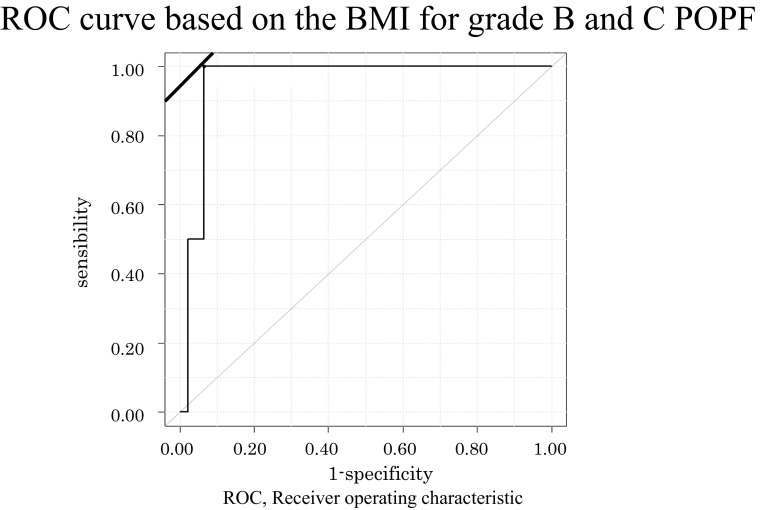
The receiver operating characteristic (ROC) *curve* based on the BMI for Grade B and C pancreatic fistula after DP using a triple-row stapler. The area under the curve = 0.957

Seventy-five patients who underwent DP were classified into a POPF Grade B/C group and none or a POPF Grade A group. Table 1 shows a comparison of the two groups regarding the patient characteristics. Seven patients (9.3%) had POPF Grade B/C and 68 patients (90.7%) had none or POPF Grade A. According to a univariate analysis, patients who had POPF Grade B/C were significantly younger (56.4 ± 5.6 vs 67.0 ± 1.5, *p* = 0.041) and had a higher BMI (26.8 ± 0.5 vs 21.4 ± 0.4 kg/m^2^, *p* = 0.0001) than the patients who had none or POPF Grade A. There were no significant differences in sex, hemoglobin A1c, or the histopathological diagnosis. The receiver operating characteristic (ROC) indicated that an age less than 57 years old and a BMI of more than 25.7 kg/m^2^ were the cut-off values for predicting POPF Grade B/C (Fig. 1). According to a multivariate analysis, a high BMI was the only significant indicator for POPF Grade B/C.

### Intra and perioperative findings

Table 2 shows a comparison of the two groups regarding the intraoperative findings. The type of operation, operative time, blood loss, RBC transfusion, thickness of the stump, or width of the stump were not significant factors according to both univariate and multivariate analyses.

Table 3 shows the correlation between the BMI and Age or thickness of the stump. No correlation was found between the BMI and Age or thickness of the stump.

**Table 3:** Correlation between the BMI and Age or the thickness of the pancreas

	BMI (kg/m^2^) ≥25.7 (*n* = 9)	BMI (kg/m^2^) <25.7 (*n* = 66)	Univariate analysis *p*
Age	62.0 ± 5.3	65.8 ± 1.7	0.457
Thickness of the stump	14.9 ± 1.5	12.5 ± 0.5	0.083

The amylase level of drainage fluid (D-Amy) was higher in the POPF Grade B/C group than in the none or POPF Grade A group. However, there were no significant differences between the two groups. D-Amy of more than 860 IU/ml was the cut-off value for predicting POPF Grade B/C on POD 3.

### Risk factors for POPF

As Table 4 shows, 5 cases of Grade B/C were detected among the 20 patients with an age ≤57, and only 2 cases were detected among the 55 patients with an age >57. In addition, 6 cases of Grade B/C were detected among 9 patients with a BMI ≥ 25.7 kg/m^2^, and only 1 case was detected among the 66 patients with a BMI < 25.7. Furthermore, 6 cases of Grade B/C were detected among the 25 patients with D-Amy ≥860 IU/ml on POD 3. As a result of the analysis, the significant risk factors for POPF after DP with using a triple-row stapler were an age ≤57 (*p* = 0.005), a BMI ≥ 25.7 kg/m^2^ (*p* < 0.0001), and D-Amy ≥860 IU/ml on POD 3 (*p* = 0.002).

**Table 4:** Analysis of the risk factors for POPF Grade B/C

	Pancreatic fistula		Univariate analysis
	*n*	Events	*p*
Age ≤57	20	5	0.005
Age >57	55	2	
BMI (kg/m^2^) ≥25.7	9	6	<0.0001
BMI (kg/m^2^) <25.7	66	1	
D-Amy day 3 ≥860 IU/ml	25	6	0.002
D-Amy day 3 <860 IU/ml	50	1	

*D*-*Amy* amylase level in drainage fluid

## Discussion

The risk factors for POPF after DP using several techniques have been previously reported. Indeed, sex [8, 23], age [4, 24, 25], BMI [5, 8, 23, 25], diabetes mellitus [12, 25], the cartridge size [26], pancreatic thickness [1, 2, 4, 23, 25], chronic pancreatitis [27], extended lymphadenectomy [24], additional organ resection [8], and duration of operation [9, 12, 28] were described as risk factors. Since the risk factors for POPF after DP using a triple-row stapler have not yet been elucidated, the risk factors for POPF Grade B/C were determined in this study.

At first, a BMI ≥ 25.7 kg/m^2^ was found to be a significant risk factor. It was previously reported that obesity is characterized as a risk factor for surgical morbidity in pancreatic resection [29, 30]. This cause may be due to the technical difficulty associated with obese patients. Although the sample size is too small to discuss the relationship between BMI and POPF in our study, the BMI may have some influence on the physiological condition of the pancreas, such as fibrosis. Next, an age ≤57 years old was also a significant risk factor. To our knowledge, a younger age has been reported to be a risk factor for POPF after DP using a double-row stapler [4, 24]. An impaired exocrine function reduces the rate of POPF in elderly patients [24, 31].

Recent studies have emphasized the importance of the early postoperative drain amylase values for predicting the occurrence of POPF [4, 7, 32]. Indeed, in the current study, D-Amy ≥860 IU/ml on POD 3 was also an independent risk factor for POPF. Alternatively, it is recommended that the drain should be removed as early as possible to reduce the incidence of intra-abdominal infections [33, 34]. Indeed, it was recently reported that the early removal of the drain significantly reduced the incidence of intra-abdominal infections which is a leading cause of POPF [33, 34]. Thus, these results suggest that removing the drain in the early perioperative phase may reduce POPF by prevention intraabdominal infection. Therefore, it is important to identify various criteria to determine the optimal time to remove the drain.

It was recently reported that the thickness of the pancreas from preoperative CT is one independent risk factor for POPF [1, 2, 4, 23, 35]. In this study, the thickness of pancreas was not a significant risk factor for POPF. At our institution, the height of the stapler was selected according to the pancreatic thickness on the cutting line by intraoperative ultrasonography. Using this method, the pancreatic thickness can be measured both easily and accurately. It is important to completely close both the main pancreatic and branch pancreatic ducts on the cut surface to prevent POPF. By selecting the optimal cartridge size and using the parenchymal flattening technique [36], POPF due to pancreatic damage when using a stapler could thus be reduced in this study. In contrast, Kleeff et al. described that mechanical stapling could crush the pancreas parenchyma, thus leading to the subsequent leakage of pancreatic juice from the branch pancreatic duct and later resulting in POPF [9]. Therefore, the mechanical jaw of the stapler should be closed gently and pancreas should be cut slowly to avoid causing any tissue damage. Furthermore, the stapler should not be released immediately after firing [36]. In this study, we experienced one case of damage to the pancreatic parenchyma at the stapling site and one case of bleeding at the staple line. Both cases occurred at the edge of the pancreatic parenchyma. We sutured that part in both cases and those two cases did not develop POPF. Taken together, the method using the triple-row stapler with our surgical procedure is thus considered to be useful for reducing POPF.

## Conclusion

To confirm the results of this study, a multicenter controlled trial using the same technique is necessary to analyze the true risk factors and thereafter establish definitive criteria to prevent the incidence of POPF after DP using a mechanical triple stapler.
